# Sensitivity and specificity of two dried blood spot methods for HIV-1 viral load monitoring among patients in Hanoi, Vietnam

**DOI:** 10.1371/journal.pone.0191411

**Published:** 2018-01-18

**Authors:** Todd M. Pollack, Hao T. Duong, Phuong T. Truong, Thuy T. Pham, Cuong D. Do, Donn Colby

**Affiliations:** 1 The Partnership for Health Advancement in Vietnam, Beth Israel Deaconess Medical Center, Hanoi, Vietnam; 2 Department of Medicine, Beth Israel Deaconess Medical Center, Boston, Massachusetts, United States of America; 3 Department of Microbiology, Bach Mai Hospital, Hanoi, Vietnam; 4 Department of Infectious Diseases, Bach Mai Hospital, Hanoi, Vietnam; 5 Center for Applied Research on Men and Community Health, Ho Chi Minh City, Vietnam; 6 SEARCH, Thai Red Cross AIDS Research Centre, Bangkok, Thailand; Institut pediatrik,Hospital Kuala Lumpur, MALAYSIA

## Abstract

The use of dried blood spot (DBS) specimens for HIV viral load (VL) monitoring is recommended to support the roll-out of routine VL monitoring in low and middle income countries (LMICs). To better understand the use of DBS for VL monitoring, we evaluated two DBS testing methods, Roche TaqMan® Free Virus Evolution protocol (DBS-FVE) and Roche TaqMan® SPEX protocol (DBS-SPEX)) in patients receiving ART at an HIV clinic in Hanoi, Vietnam. Sensitivity, specificity, positive predictive value (PPV), and negative predictive value (NPV) were calculated for each DBS testing method at the thresholds of 1000 and 5000 copies/ml compared to plasma VL. At a threshold of 1000 copies/ml, sensitivity, specificity, PPV and NPV of the DBS-SPEX method were 98.8% (95% CI: 93.3%-100%), 74.3% (95% CI: 70.8%-77.5%), 31.5% (95% CI: 25.8%-37.6%), and 99.8% (95% CI: 98.9%-100%), respectively. Increasing the VL threshold value to 5000 copies/ml improved specificity (97.9% CI: 96.6%-98.9%) and PPV (83.9% CI: 74.5%-90.9%). Using the DBS-FVE method, at the threshold of 1000 copies/ml and with a correction factor of +0.3 log copies/ml, sensitivity was 95.1% (87.8%-98.6%) and specificity was 98.8% (97.7%-99.5%). Sensitivity decreased at the threshold of 5000 copies/ml (65.8%, 95% CI: 54.3%-76.1%). With a correction factor of +0.7 log copies/ml, the sensitivity was 96.3% (89.6%-99.2%) and specificity was 98.2% (96.9%-99.1%) at the threshold of 1000 copies/ml. We found that the Roche DBS-FVE method, with a +0.7 log copies/ml correction factor, performed well with sensitivity and specificity greater than 96% at a VL threshold of 1000 copies/m. These findings add to the growing body of evidence supporting the use of DBS VL testing for ART monitoring. Future research should evaluate the association between VL results by DBS and clinical outcome measures such as HIV drug resistance, morbidity, and mortality.

## Introduction

HIV viral load (VL) testing is recommended by the World Health Organization (WHO) as the preferred method for monitoring treatment response and diagnosing treatment failure among patients on antiretroviral therapy (ART) [1]. VL monitoring has higher sensitivity and positive predictive value (PPV) for the diagnosis of treatment failure compared to immunologic or clinical monitoring [2]. VL monitoring enables accurate and early detection of virological failure allowing patients to switch regimens before the accumulation of drug resistance mutations [3]. VL confirmation of immunologic or clinical treatment failure also prevents inappropriate switching to significantly more expensive second-line regimens. In their latest HIV Guidelines, WHO recommended the phasing in of routine VL monitoring (testing all patients at 6 months after ART initiation, and then at least every 12 months) in low and middle income countries (LMICs) [1].

Following WHO guidance, many LMICs are scaling up HIV VL testing, moving from a strategy of targeted testing to annual routine VL for all patients on ART. However, countries face numerous challenges to scaling up HIV VL testing including lack of sufficient funding, limited laboratory infrastructure, shortage of skilled clinical and laboratory staff, lack of understanding of the value of VL monitoring among clinicians and patients, weak specimen transport systems, and inefficient systems for result feedback [4,5]. In 2015, only 22% of patients on ART globally (86 countries reported data) received a VL test [6]. At the current rate, the scale-up of VL testing will not meet the demand by 2020 [6].

Dried blood spots (DBS) specimens are increasingly being used in the scale-up of HIV VL testing in Africa and Asia. DBS specimens are easier to collect, store, and transport than plasma samples [7]. DBS can be collected through finger-prick, eliminating the need for phlebotomy and centrifugation [8]. DBS specimens, once dried, are not considered biohazardous and they can be stored and shipped at ambient temperature, eliminating the need for cold chain transport [1,9]. Without a need for centrifugation, frozen shipment, or highly trained staff, DBS can greatly simplify storage and transport to a reference laboratory from remote sites and, thus, can overcome the lack of laboratory capacity in resource limited settings.

Despite these advantages, the best approach for monitoring ART using DBS is still being defined. The appropriate threshold for defining virological failure using DBS remains a topic of debate [10]. The WHO, based on findings of a systematic review of 43 studies, recently recommended a threshold for detection of treatment failure using DBS of 1000 copies/ml [1]. However, the sensitivity and specificity of DBS varies depending on the VL platform. In particular, the specificity of DBS testing at lower levels of viremia has been questioned as DBS may overestimate the VL compared to plasma through the detection of cell-associated HIV RNA [9]. Furthermore, many studies examining the performance of DBS testing have been performed using venous whole blood specimens prepared in the laboratory under controlled circumstances rather than based on specimens obtained in clinical settings [1].

Vietnam has approximately 262,000 people living with HIV in 2017 [11] and more than 110,000 patients currently receiving ART [12]. A recent survey found that 7% of patients on ART in Vietnam had detectable VL above 1,000 copies/ml [13]. A phased implementation of routine HIV VL monitoring for patients on ART has recently started [14]. To better understand the use of DBS for ART monitoring, we integrated DBS testing using two Roche TaqMan® DBS methods into the ongoing Viral Load Monitoring in Vietnam (VMVN) study. VMVN is a prospective, randomized controlled trial of VL monitoring vs standard (CD4) monitoring in a patient population starting ART in Vietnam. The study was described in detail elsewhere [15]. We aimed to investigate the correlation between VL values obtained from DBS specimens with those from plasma specimens and to test associations with clinical outcome measures such as plasma viral suppression, resistance, morbidity, and mortality. This paper reports sensitivity and specificity of two DBS testing methods (Roche TaqMan® Free Virus Evolution protocol (DBS-FVE) and Roche TaqMan® SPEX protocol (DBS-SPEX)) compared to plasma VL at the threshold of 1000 and 5000 copies/ml.

## Methods

### Patients and samples

DBS specimens were obtained from the participants being enrolled in the VMVN study, a prospective, randomized controlled trial of routine VL monitoring versus standard monitoring in a patient population starting ART between April 2011 and April 2014. The intervention group received VL testing every six months in addition to the standard monitoring approach of CD4 count testing every six months and targeted VL testing to confirm suspected treatment failure based on the presence of clinical and/or immunological criteria [16]. Those who were still in active follow-up in 1/2015 received a DBS specimen at the time of their scheduled plasma VL. The DBS collection was conducted in participants on both the standard monitoring and VL monitoring study arms. As all subjects in the VMVN study were already on ART at the time of the first DBS specimen collection, we enrolled an additional 79 ART-naïve patients who had longitudinal DBS testing starting from the baseline (pre-ART).

Here we present data from all subjects with corresponding plasma and DBS VL at all time periods available up to November 25, 2016. The sample size was considered sufficient for a method comparison as described in the Clinical Laboratory Standards Institute guidelines, CLSI EP9-A2 [17]. The guidelines state that a sample size of n = 40 is used to determine the correlation of two test methods, and the sample size is multiplied by the number of subgroups that are analyzed.

The study was approved by the Institutional Review Board of Beth Israel Deaconess Medical Center (#2010P000334) in Boston, USA and the Ethical Committee of Bach Mai Hospital in Hanoi, Vietnam. All subjects provided written informed consent prior to study participation. The VMVN study was registered at www.clinicaltrials.gov (ClinicalTrials.gov identification number: NCT01317498). The study was conducted in accordance with the principles of the Declaration of Helsinki.

### Specimen collection and preparation

Whole blood samples were collected via venipuncture at each time period for both plasma and DBS testing. Samples for plasma VL measurement were transferred from the HIV clinic to the hospital’s microbiology lab. For preparation of the DBS specimen, immediately after collection, 1 drop of blood was applied directly from the syringe onto each of the 5 spots on a Whatman 903® card. To approximate real-world conditions, no precise measurement or calibration was performed, but an estimated 70 μL of blood was necessary to fill each spot on the card. Spots were dried at room temperature for a minimum of 4 hours. Following drying, DBS specimens were either processed for VL testing or were frozen for future testing. Samples were kept at 4–8 degrees Celsius and either tested within two weeks (mean time: 6.9 days, 95% CI: 6.6–7.3 days) or stored for future testing at -70 degrees Celsius. Prior to freezing, specimens for storage were labeled and placed in a gas-impermeable bag containing 2 desiccant packs per card.

### Viral load measurement on plasma

The plasma sample was processed using the COBAS® AmpliPrep/COBAS® TaqMan® HIV-1 Test v2.0 kit (Roche Molecular Systems, Branchburg, NJ). Using protocol HI2CAP96 and following the manufacturer’s recommendation, the plasma input volume was 1.0 mL, the nucleic acid extraction product was 65 μL, and the volume used for real-time PCR was 50 μL.

### Viral load measurement on dried blood spot

#### Dried fluid spot procedure using SPEX buffer (DBS-SPEX)

Following the manufacturer’s instructions, one blood spot of 70 μL was incubated with 1mL of a Guanidinium-based sample pre-extraction (SPEX) buffer in a thermomixer set to 56 degrees Celsius and 1000 rpm continuous shaking for 10 minutes. The sample was then processed using the COBAS® AmpliPrep/COBAS® TaqMan® HIV-1 Test v2.0 (Roche Molecular Systems, Branchburg, NJ) and the dried fluid spot procedure protocol (HI2DFSP96) per the package insert instructions.

#### Free Virus Elution method (DBS-FVE)

Following the manufacturer’s instructions, one blood spot of 70 μL was incubated in 1 mL of calcium and magnesium free-phosphate-buffered saline (PBS) in an S-tube at room temperature for 60 minutes without shaking. The PBS eluate, with the DBS paper, was processed using the COBAS® AmpliPrep/COBAS® TaqMan® HIV-1 Test v2.0 (Roche Molecular Systems, Branchburg, NJ), and the dried fluid spot procedure protocol (HI2DFSP96) per the package insert instructions. A correction factor was added to each result obtained via the FVE method. We compared the use of a correction factor of +0.3 log copies/ml as recommended by Wu et al. [18] with a correction of +0.7 log copies/ml as recommended by the U.S. Centers for Disease Control and Prevention [19].

### Statistical analysis

The distribution of VL results from each testing method is presented. Undetectable results were replaced with the midpoint value between 0 and the lower limit of detection; for plasma, a value of 9 copies/ml (limit of detection <20 copies/ml) and, for DBS, a value of 199 copies/ml (limit of detection < 400 copies/ml) was used. For results above the limit of detection (>10,000,000 copies/ml), a value of 10,000,001 copies/ml was used. Sensitivity, specificity, PPV, and negative predictive value (NPV) with 95% confidence intervals (95% CI) were calculated for each DBS testing method at the thresholds of 1000 and 5000 copies/ml compared to gold standard plasma VL. All analyses were performed using Stata/SE 14.0 (Stata Corporation, College Station, TX).

## Results

There were 757 specimens from 436 patients tested for HIV-1 VL using three methods: plasma, DBS-SPEX, and DBS-FVE. Mean age of these patients was 35.2 years old (range: 18.5–74.1), and 60.8% were male. Of the 436 patients, 161 (36.9%) had more than one specimen (range: 2–4) collected at different time points of ART. Of the 757 specimens tested, 79 were collected before ART (10.5%), 32 (4.2%) at 6 months, 69 (9.1%) at 12 months, 69 (9.1%) at 18 months, 91 (12.0%) at 24 months, 122 (16.1%) at 30 months, and 295 (39.0%) at 36 months after ART initiation. Approximately one third of the DBS specimens were tested within 2 weeks (34.2%), the remaining (65.8%) were tested after being stored at –70°C.

The distribution of actual VL values of each testing method is presented in Table 1. A large proportion of samples (81.0%) had an undetectable plasma VL (below 20 copies/ml), all from those who were already on ART. Only 10.7% (n = 81) had a plasma VL at or above 1000 copies/ml, and most of them (n = 77, 95.0%) were from those who were not yet started on ART. About one third (n = 254) of the 757 specimens had a DBS-SPEX VL at or above 1000 copies/ml, and approximately 70% of them were already on ART for at least six months (n = 177). The percentage of specimens with a DBS-FVE VL at or above 1000 copies/ml was lower (n = 69, 9.1%), and most of them (n = 65, 94.2%) were from those who were not on ART. About half of the DBS-SPEX VL results (45.4%) and more than two third of the DBS-FVE VL results (88.1%) were below the limit of detection (< 400 copies/ml), most of which were from patients on ART.

**Table 1:** Distribution of actual viral load values of each testing method.

		Total (n = 757) n (column %)	Before ART (n = 79) n (row %)	On ART (n = 678) n (row %)
**Plasma VL copies/ml**				
VL<20		613 (81.0%)	0	613 (100.0%)
20≤VL<1000		63 (8.3%)	2 (3.2%)	61 (96.8%)
1000≤VL<5000		2 (0.3%)	1 (50.0%)	1 (50.0%)
≥5000		79 (10.4%)	76 (96.2%)	3 (3.8%)
**DBS-SPEX VL copies/ml**				
VL<400		344 (45.4%)	2 (0.6%)	342 (99.4%)
400≤VL<1000		159 (21.0%)	0	159 (100.0%)
1000≤VL<5000		167 (22.1%)	6 (3.6%)	161 (96.4%)
≥5000		87 (11.5%)	71 (81.6%)	16 (18.4%)
**DBS-FVE VL copies/ml**				
VL<400		667 (88.1%)	4 (0.6%)	663 (99.4%)
400≤VL<1000		21 (2.8%)	10 (47.6%)	11 (52.4%)
1000≤VL<5000		27 (3.6%)	24 (88.9%)	3 (11.1%)
≥5000		42 (5.5%)	41 (97.6%)	1 (2.4%)

Sensitivity, specificity, PPV and NPV of the VL testing from DBS compared to plasma VL testing for detecting virological treatment failure are presented in Tables 2 and 3. At a threshold of 1000 copies/ml, sensitivity and NPV of the DBS-SPEX method were high: 98.8% (95% CI: 93.3%-100%), and 99.8% (95% CI: 98.9%-100%), respectively; but specificity and PPV were much lower: 74.3% (95% CI: 70.8%-77.5%), and 31.5% (95% CI: 25.8%-37.6%), respectively. As expected, increasing the VL threshold value to 5000 copies/ml improved specificity and PPV (Table 2).

**Table 2:** Sensitivity (Se) and specificity (Sp) of the DBS-SPEX compared to plasma.

Threshold	Plasma VL ≥ Threshold	Plasma VL < Threshold
**1000 copies/ml**		
DBS VL ≥ Threshold	80	174
DBS VL < Threshold	1	502
Se, Sp	Se: 98.8% (93.3%-100%)	Sp: 74.3% (70.8%-77.5%)
PPV, NPV	PPV: 31.5% (25.8%-37.6%)	NPV: 99.8% (98.9%-100%)
**5000 copies/ml**		
DBS VL ≥ Threshold	73	14
DBS VL < Threshold	6	664
Se, Sp	Se: 92.4% (84.2%-97.2%)	Sp: 97.9% (96.6%-98.9%)
PPV, NPV	PPV: 83.9% (74.5%-90.9%)	NPV: 99.1% (98.1%-99.7%)

**Table 3:** Sensitivity (Se) and specificity (Sp) of the DBS-FVE compared to plasma.

	Correction factor + 0.3 log copies/ml		Correction factor + 0.7 log copies/ml	
Threshold	Plasma VL ≥ Threshold	Plasma VL < Threshold	Plasma VL ≥ Threshold	Plasma VL < Threshold
**1000 copies/ml**				
DBS VL ≥ Threshold	77	8	78	12
DBS VL < Threshold	4	668	3	664
Se, Sp	Se: 95.1% (87.8%-98.6%)	Sp: 98.8% (97.7%-99.5%)	Se: 96.3% (89.6%-99.2%)	Sp: 98.2% (96.9%-99.1%)
PPV, NPV	PPV: 90.6% (82.3%-95.8%)	NPV: 99.4% (98.5%-99.8%)	PPV: 86.7% (77.9%-92.9%)	NPV: 99.6% (98.7%-99.9%)
**5000 copies/ml**				
DBS VL ≥ Threshold	52	0	67	2
DBS VL < Threshold	27	678	12	676
Se, Sp	Se: 65.8% (54.3%-76.1%)	Sp: 100.0% (99.5%-100.0%)	Se: 84.8% (75.0%-91.9%)	Sp: 99.7% (98.9%-100.0%)
PPV, NPV	PPV: 100.0% (93.2%-100.0%)	NPV: 96.2% (94.5%-97.5%)	PPV: 97.1% (89.9%-99.6%)	NPV: 98.3% (97.0%-99.1%)

At the treatment failure threshold of 1000 copies/ml and with a correction factor of +0.3 log copies/ml, sensitivity, specificity, PPV, and NPV of the DBS-FVE method were all high, above 90% (Table 3). The sensitivity was 95.1% (87.8%-98.6%); four specimens with the plasma VL ≥ 1000 copies/ml (range: 7,000–23,700 copies/ml) were misclassified by the FVE-DBS (result < 1000 copies/ml: three cases <400 copies/ml, and one case 962 copies/ml). Of these four, two were obtained pre-ART, and the other two were obtained at 24 months and at 36 months of ART. The specificity was 98.8% (97.7%-99.5%) with eight specimens misclassified as having VL ≥1000 copies/ml according to DBS-FVE (range: 1010–4210 copies/ml). All eight specimens were from patients who were on ART for at least six months and had a plasma VL below 60 copies/ml. Sensitivity decreased substantially at the threshold of 5000 copies/ml (65.8%, 95% CI: 54.3%-76.1%), but there was little change in other parameters (specificity, PPV, and NPV).

Using the correction factor of +0.7 log copies/ml slightly increased the sensitivity of the DBS-FVE method, and slightly decreased PPV for the threshold of 1000 copies/ml. Specifically, sensitivity increased from 95.1% (+ 0.3 log copies/ml) to 96.3% (+ 0.7 log copies/ml), and PPV decreased from 90.6% (+ 0.3 log copies/ml) to 86.7% (+ 0.7 log copies/ml). Similarly, at the threshold of 5000 copies/ml sensitivity increased moderately from 65.8% (+ 0.3 log copies/ml) to 84.8% (+ 0.7 log copies/ml), but there was little change in other parameters (specificity, PPV, and NPV).

## Discussion

HIV-1 VL testing by DBS has many advantages over plasma VL testing and is recommended for LMIC to support the scale-up of routine HIV VL [20]. Several DBS testing platforms are available, each with its own testing characteristics. In this study among a cohort of patients in Vietnam, we evaluated two Roche Taqman® DBS methods compared to standard plasma VL testing. Sensitivity and specificity varied based on the DBS testing method, the correction factor applied to the DBS-FVE method, and the VL threshold.

At a threshold of 1,000 copies/ml, the sensitivity and NPV of DBS-SPEX was high at 98.8% (95% CI: 93.3%-100%) and 99.8% (95% CI: 98.9%-100%), respectively. However, the specificity and PPV were much lower, 74.3% (95% CI: 70.8%-77.5%), and 31.5% (95% CI: 25.8%-37.6%), respectively. Our findings suggest that the DBS-SPEX method overestimated the VL. About one fourth of the 254 specimens with a plasma VL below 1000 copies/ml had a DBS-SPEX result at or above 1000 copies/ml. Thus using the DBS-SPEX result would lead to unnecessary VL retesting or switching to second–line ART in accordance with the current Vietnam Ministry of Health guidelines for HIV/AIDS care and treatment [14]. These findings are also consistent with the recent WHO review which reported a pooled estimated sensitivity of the Roche DBS-SPEX of 99% (97%-100%) and specificity of 44% (18–74%) at 1000 c/mL [1]. Due to the poor specificity, the SPEX method is no longer recommended [20].

We found high sensitivity, specificity, PPV, and NPV of the DBS-FVE testing method compared to plasma VL at a threshold of 1,000 copies/ml. Using the correction factor of +0.3 log copies/ml, as recommended by Wu et al. [18], sensitivity, specificity, PPV, and NPV were all > 90%. Applying a correction factor of +0.7 log copies/ml, as recently recommended by the US Centers for Disease Control and Prevention [19], increased the sensitivity from 95.1% (+ 0.3 log copies/ml) to 96.3% (+ 0.7 log copies/ml) and reduced the number of misclassified positive specimens from four to three (plasma VL ≥ 1,000 copies/ml, but DBS-FVE <1,000 copies/ml). However, the specificity decreased marginally from 98.8% (+ 0.3 log copies/ml) to 98.2% (+ 0.7 log copies/ml), increasing the number of misclassified negative specimens from eight to 12 (plasma VL < 1,000 copies/ml, but DBS-FVE ≥ 1000 copies/ml). Due to the relatively low number of plasma VLs above 1000 copies/ml in our sample, the small reduction in the number of misclassified positive cases when using the higher correction factor likely underestimates the benefit of the higher sensitivity with the +0.7 log copies/ml correction factor. In addition, the loss of specificity may be less important as, according to treatment guidelines, VL results above the treatment failure threshold need to be confirmed with repeat VL testing following one to three months of adherence counseling.

The Roche DBS-FVE method has been evaluated in several previous studies. In one study by Taieb et al., also conducted in Vietnam, which compared the DBS-FVE method (correction factor +0.3 log copies/ml) with the Abbott RealTime assay, the sensitivity of DBS-FVE at a plasma threshold of 1,000 copies/ml was significantly lower than in our study (55%, 95% CI: 42%-69%) [21]. Perhaps differences in sample collection, storage and laboratory conditions between the two studies could, at least partially, explain the different findings. For example, we used Whatman 903 filter cards in our study where as Taieb et al. utilized Munktell TFN cards. However, this is unlikely to be completely responsible for difference in sensitivity as one previous study validated and reported that DBS collected on the Munktell TFN filter papers performed similarly to DBS collected on W-903 filter paper for quantitative VL analysis and HIV drug resistance (HIVDR) detection [22]. Both Taieb and our study utilized whole blood samples collected by phlebotomy, but in the Taieb study the whole blood was deposited onto the filter cards in the laboratory using a calibrated pipette, whereas in our study the filter cards were prepared immediately following phlebotomy inside the HIV clinic by the nurse phlebotomist. Storage conditions prior to testing also differed between the two studies. In the Taieb study, DBS specimens were kept for two weeks at room temperature and then frozen at -20 degrees, whereas specimens in our study were preserved at 4–8 degrees Celsius and then, within two weeks, were either tested or transferred to storage at –70 degrees Celsius. One additional previous evaluation of the DBS-FVE method, conducted by Roche using the +0.3 log copies/ml correction factor, found a sensitivity of 90%, similar to our findings [18]. Finally, the systematic review conducted by WHO reported moderately high sensitivity (85%, 95% CI: 77%-91%) and specificity (94%, 95% CI: 85%-98%) of the DBS-FVE method [1]. However, whether or not a correction factor was applied in those studies is unclear.

Using a VL threshold of 5,000 copies/ml, the specificity of the DBS-FVE method improved but the sensitivity was substantially lower. A recent analysis using routine data from Kenya to calculate the rate of misclassification with different DBS platforms concluded that the more stringent VL threshold of 1000 copies/ml, compared to 5000 copies/ml, would lead to an increase in unnecessary repeat testing and regimen switches [23]. Using our data, however, we found an overall misclassification rate of 1.6% using a threshold of 1000 copies/ml, compared to a misclassification rate of 3.6% at a threshold of 5000 copies/ml using the correction factor of +0.3 log copies/ml.

Our study has several limitations. Although the study was conducted as part of a randomized controlled study, we attempted to approximate real-world clinical conditions. DBS cards were prepared in the HIV clinic at the time of phlebotomy and the cards were dried in the HIV clinic before transfer to the laboratory. However, the clinic and laboratory are located within a tertiary care hospital in Hanoi and therefore conditions for collection, storage and shipment will differ compared to more remote settings in Vietnam. Specifically, DBS specimens in our study were stored prior to testing at either 4–8 degrees (34.2%) or –70 degrees Celsius (65.8%). As the performance of DBS testing may be affected by a delay between collection and VL measurement, our findings may not be applicable under different conditions of specimen handling and storage. This is particularly important since implementation of DBS testing in remote areas of Vietnam would likely require DBS specimens to be stored at room temperature or under refrigeration before being shipped to a central laboratory. Finally, the majority of samples in our study were from patients who were already on ART for at least six months (89.5%), thus a large proportion (81.0%) had an undetectable plasma VL (below 20 copies/ml). The fact that we found DBS-FVE to be more reliable than the DBS-SPEX should be reconfirmed in other populations with different distributions of VL.

## Conclusion

HIV VL testing by DBS is recommended to support the scale up VL testing in LMIC. The sensitivity and specificity of DBS varies depending on the VL platform. We found that the Roche DBS-FVE method, with a +0.7 log copies/ml correction factor, out-performed the Roche DBS-SPEX method, and had a sensitivity and specificity greater than 96% at a VL threshold of 1000 copies/ml. These findings add to the growing body of evidence supporting the use of DBS VL testing for ART monitoring. Future research should evaluate the association between VL results by DBS and clinical outcome measures such as HIVDR, morbidity, and mortality.

## Supporting information

S1 Raw Data (DTA)
